# Single-pill combinations: a therapeutic option or necessity for vascular risk treatment?

**DOI:** 10.3109/21556660.2013.801605

**Published:** 2013-05-07

**Authors:** Niki Katsiki, Vasilios G. Athyros, Asterios Karagiannis

**Affiliations:** Second Propedeutic Department of Internal Medicine, Medical School, Aristotle University of Thessaloniki, Hippocration Hospital, ThessalonikiGreece

**Keywords:** Anti-hypertensive, Anti-platelet drugs, Hypolipidemic, Hypoglycemic, Single-pill combinations, Vascular risk

In a recently published paper in the *Journal of Drug Assessment*, Axthelm *et al.*1 reported on the effectiveness of single-pill combination (SPC) aliskiren 300 mg/amlodipine 10 mg in high-risk subgroups of hypertensive patients with uncontrolled blood pressure (BP). Briefly, 4-week’s treatment with SPC aliskiren 300 mg/amlodipine 10 mg resulted in further lowering of both systolic and diastolic BP in elderly (≥65 years), overweight/obese (body mass index ≥25 kg/m^2^) and diabetic patients as well as individuals with at least one metabolic risk factor (i.e., serum glucose ≥5.56 mmol/l, low density lipoprotein cholesterol ≥4.16 mmol/l or triglycerides ≥2.28 mmol/l) that were inadequately controlled by prior use of SPC olmesartan 40 mg/amlodipine 10 mg1. The efficacy and safety of aliskiren/amlodipine SPCs in patients previously on either drug monotherapy were also reported in earlier studies. Of note, adverse effects such as peripheral edema as well as discontinuation rates were fewer in the SPC groups2–4.

Cardiovascular disease (CVD) represents the main cause of death worldwide and thus research still focuses on potential genetic and physiological biomarkers, imaging techniques, healthcare technologies and indices for both CVD prevention and treatment as well as personalized prediction models5. There are several CVD risk factors, including hypertension, dyslipidemia, diabetes mellitus (DM), smoking and obesity, as well as platelet dysfunction. Certain drugs are currently available for treating these risk factors, whereas drug combinations are frequently needed to achieve therapeutic goals especially in hypertension, DM and coronary heart disease (CHD).

With regard to hypertension, the 2009 reappraisal of the European guidelines (European Society of Cardiology/European Society of Hypertension)6 recommends the use of a renin–angiotensin–aldosterone system (RAAS) blocker plus calcium channel blocker (CCB) or RAAS blocker plus diuretic or CCB plus diuretic as possible two-drug combination therapies. Such combinations are available as SPCs7. For example, the first SPC of an angiotensin receptor blocker (ARB) and a CCB was valsartan plus amlodipine which, apart from achieving better efficacy than each component, was also shown to significantly decrease the risk of edema, a frequent side-effect of dihydropyridine CCBs8. Similarly, olmesartan has been combined with either amlodipine or hydrochlorothiazide in SPCs9, as is the case with telmisartan10,11, losartan12,13, irbesartan14–16, candesartan17,18 and aliskiren (a direct renin antagonist)2,3,19.

Perindopril, an angiotensin converting enzyme (ACE) inhibitor, and amlodipine SPC can be also used to adequately treat hypertensives20, whereas perindopril/indapamide fixed-dose combination is effective in reducing both macro- and micro-vascular diabetic complications21,22. Another therapeutic option is SPCs of benazepril (ACE inhibitor) plus amlodipine or hydrochlorothiazide; the former combination decreased the progression of chronic kidney disease to a greater extent compared with the latter23. Amlodipine is also available in a fixed-dose combination with hydrochlorothiazide24.

It should be noted that the combination of an ACE inhibitor with an ARB is currently not recommended based on the results of the Ongoing Telmisartan Alone and in Combination with Ramipril Global Endpoint Trial (ONTARGET) study where more adverse effects were reported in the combination group than monotherapy groups25. However, a recent study by the ONTARGET investigators26 showed that ramipril (ACE inhibitor) and telmisartan combination did not raise the rate of stroke, CVD or renal events in patients with DM compared with monotherapy groups.

Dual ACE inhibitor (or ARB) and aliskiren treatment is also currently not recommended based on the results of the Aliskiren Trial In Type 2 Diabetes Using Cardio-Renal Disease Endpoints (ALTITUDE)27 which was prematurely ended as it did not demonstrate the benefit predicted by the initial protocol; safety issues also presented (i.e., increased incidence of stroke, kidney dysfunction, hyperkalemia and hypotension)28. However, two other aliskiren trials are still running in patients with heart failure: the Aliskiren Trial of Minimizing OutcomeS for Patients with HEart failure (ATMOSPHERE)29 and the Aliskiren Trial on Acute Heart Failure Outcomes (ASTRONAUT)30.

The best combination therapy differs with regard to patient populations; for example, African-American individuals and patients with heart failure will benefit more from a RAAS inhibitor plus a diuretic, whereas a RAAS inhibitor combined with a CCB will produce a greater reduction of CVD risk31.

SPCs of three antihypertensive drugs are also commercially available including ARBs or aliskiren combined with amlodipine and hydrochlorothiazide32,33. Taking into consideration that almost one-fifth of hypertensive patients will require three antihypertensive drugs to achieve BP goals, this triple fixed-dose combination therapy appears rational34 with several beneficial effects in terms of compliance, clinical outcomes and economics35.

With regard to hypolipidemic drugs, the first fixed-dose combination includes simvastatin and ezetimibe36, a useful therapeutic option when lipid goals are not achieved with statin monotherapy as well as in statin intolerant patients37,38. Furthermore, SPCs of statins and fibrates have been developed. Briefly, a statin (atorvastatin, pravastatin or simvastatin) and fenofibrate fixed-dose combination may be used in patients with mixed hyperlipidemia, i.e. those with low density lipoprotein cholesterol (LDL-C) levels on target but with low high density lipoprotein cholesterol (HDL-C) or high triglycerides39–42. In such cases, residual CVD risk should be adequately treated43–45.

Of note, atorvastatin has been also combined with metformin or sitagliptin with similar efficacy and safety as the individual components46,47 as well as with amlodipine with this SPC enabling more patients to reach LDL-C and BP targets than single-agent or placebo therapy48. Another SPC includes simvastatin and extended release (ER) niacin and it was more effective in lipid-lowering than monotherapies with similar safety profile49. A fixed-dose combination of lovastatin and ER niacin has been also evaluated50. Of note, a polypill containing a statin, niacin and aspirin has been previously suggested for treating mixed hyperlipidemia51.

In the field of hypoglycemic drugs, SPCs of metformin and dipeptidyl peptidase (DPP)-IV inhibitors (i.e., sitagliptin, vildagliptin and saxagliptin) are frequently used in daily practice as they achieve sufficient glycemic control with less gastrointestinal adverse events52–54. Other fixed-dose combinations include metformin and glimepiride55, metformin and pioglitazone56, metformin and repaglinide57, sitagliptin and pioglitazone58, as well as mitiglinide and voglibose59. Such SPCs were shown to improve adherence and clinical outcomes as well as reduce medical costs60,61; diabetic patients on SPCs feel also more satisfied than those taking drugs as separate formulations62.

The first available fixed-dose combination of antiplatelet drugs included acetylsalicylic acid (ASA) and extended-release dipyridamole which was both efficient and safe in atherothrombotic events prevention settings63,64. More recently, SPCs of ASA and clopidogrel have become commercially available65; their long-term effectiveness and safety remain to be established. In contrast, newer antiplatelet drugs that have proven their clinical efficacy such as prasugrel and ticagrelor66 are currently not included in SPCs. These drugs were shown to reduce non-fatal ischemic events, as well as CVD and all-cause mortality (only for ticagrelor) in acute coronary syndromes66,67 and taking into consideration that several patients may be resistant to aspirin or clopidogrel68, their role in daily practice is of particular importance in treating high-risk patients. Antiplatelet drugs may also be combined with proton pump inhibitors to reduce the risk of gastrointestinal ulcers as is the case with the SPC of ASA and esomeprazole69.

Fixed-dose combination of aspirin plus low-dose warfarin was proven insufficient to protect from thrombogenesis in patients with chronic atrial fibrillation70. Of note, novel anti-coagulant agents are now in the market (i.e., dapigatran, rivaroxaban and apixaban) and thus treatment choice should be individualized based on the more recent guidelines of several international cardiovascular societies and associations71,72. SPCs with such drugs have not yet been developed.

In general, it is more likely to achieve better compliance with the use of SPCs7, especially in patients receiving several drugs due to comorbid conditions, thus possibly reaching therapeutic targets. Furthermore, SPCs include lower doses of each drug than would be necessary to achieve goals with monotherapy, a fact that may explain their better tolerability compared with the higher dose monotherapy. However, SPCs may also have certain disadvantages such as higher cost, less flexibility in altering doses and differences in the duration of action of the combined drugs73.

The use of one polypill that will contain different drugs targeting CVD risk including a beta-blocker, diuretic, ACEi, aspirin and statin has also been suggested although long-term data are missing74–77. Such polypills are expected to increase patient compliance, thus resulting in better prevention and therapeutic outcomes78,79; patients with acute myocardial infarction represent a promising population for this treatment strategy80. Furthermore, the beneficial effects of the polypill in terms of cost effectiveness are highly tempting, especially for countries with low national incomes and economical crises81,82. However, polypills were associated with moderately more side-effects than the component drugs83, whereas a recent meta-analysis reported a moderately lower tolerability rate in patients on polypills compared with those on placebo or one component76. The results of on-going clinical trials in several countries worldwide in both primary and secondary CVD settings, also comparing the effects of the time of administration (i.e., evening vs. morning), will contribute in evaluating the clinical implications of such ‘multidrugs’84–86.

Overall, the use of SPCs seems both needed and promising in CVD prevention. However, as certain disadvantages may exist, further and larger clinical trials are required to establish their role in daily practice.

## Transparency

### Declaration of funding

None to declare.

### Declaration of financial/other relationships

None to declare; this editorial was written independently. The authors did not receive financial or professional help with the preparation of the manuscript.

## Acknowledgments

None to declare.
